# The Efficacy of Radiofrequency Ablation in Treating Nonmetastatic Medullary Thyroid Carcinoma: A Case Study

**DOI:** 10.1002/cnr2.70419

**Published:** 2025-12-10

**Authors:** Hojat Ebrahiminik, Hossein Chegeni, Haleh Chehrehgosha

**Affiliations:** ^1^ Department of Interventional Radiology and Radiation Sciences Research Center AJA University of Medical Sciences Tehran Iran; ^2^ Tirad Imaging Institute Tehran Iran; ^3^ Hazrat Rasool Hospital, School of Medicine Iran University of Medical Sciences Tehran Iran

**Keywords:** ablation, medullary thyroid carcinoma, radiofrequency ablation, thyroid

## Abstract

**Background:**

Radiofrequency ablation (RFA) can be an alternative management method for patients who are not candidates for surgery or who refuse it. There are limited data concerning the role of RFA in patients with medullary thyroid carcinoma (MTC). This case is presented to discuss the efficacy of RFA in the management of nonmetastatic MTC.

**Case:**

In this report, we present the case of an 83‐year‐old male with MTC who refused thyroidectomy. RFA was performed and he was followed for 1 year. In this case, RFA resulted in significant reductions in tumor size and calcitonin levels without any significant complications.

**Conclusion:**

This research indicates that Radiofrequency Ablation (RFA) can yield acceptable results in the management of Medullary Thyroid Cancer (MTC). However, ongoing investigation remains crucial to clarify long‐term outcomes and to compare RFA's efficacy against conventional management methods.

## 1 Background

Thyroid cancer incidence has increased significantly over the past four decades worldwide. It is now the 13th most commonly diagnosed cancer overall and ranks sixth among women [1]. Medullary thyroid carcinoma (MTC) is a rare and aggressive type of thyroid cancer. The diagnosis of thyroid carcinoma is based on fine needle aspiration (FNA) cytology; however, cytomolecular testing can aid in thyroid nodule management [2, 3]. The conventional treatment for MTC is total thyroidectomy, which may need to be repeated due to the high recurrence rate resulting in several complications. However, with the advent of minimally invasive techniques, radiofrequency ablation (RFA) has emerged as a promising alternative for treating MTC, although it is an area with limited literature [4]. RFA destroys tumor cells by generating localized heat that causes protein denaturation and cell death [5]. The rationale behind utilizing RFA in MTC treatment stems from its minimally invasive nature and potential to effectively reduce tumor size while preserving surrounding healthy tissue and also decrease the serum level of calcitonin [6]. Traditional surgical approaches carry significant risks, including complications such as vocal cord paralysis and hypothyroidism due to extensive tissue removal. In contrast, RFA targets the tumor directly, minimizing damage to adjacent structures and reducing recovery time. This is particularly advantageous for elderly patients or those with comorbidities that complicate surgical options [7].

This study presents a case study of an elderly male with MTC who underwent RFA and discusses the fact that RFA may be a safe and effective alternative treatment to surgery in MTC patients who refuse or are unsuitable for it [8]. This case is unique as it represents one of the first documented instances of RFA being employed in patients with MTC instead of surgery, highlighting its potential as a viable treatment option in settings where traditional surgery poses excessive risk. This case contributes valuable insights to the existing literature by demonstrating that RFA can be a safe and effective alternative to surgery in select MTC patients. The findings align with emerging studies suggesting that RFA not only reduces tumor volume but also preserves thyroid function, which is crucial for long‐term patient health [2, 9].

## 2 Case

**FIGURE 1:**
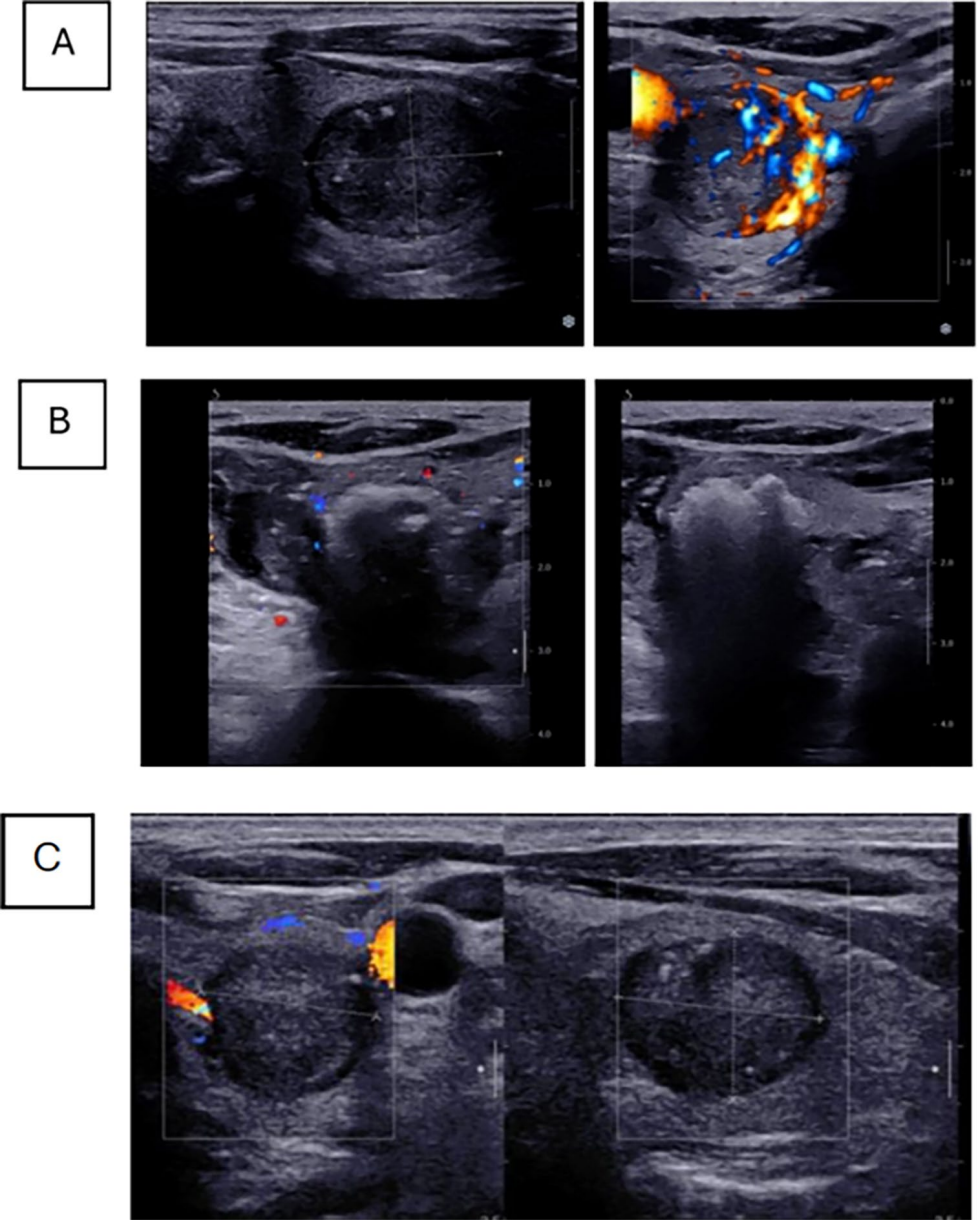
(A) Hypoechoic hypervascular left thyroid nodule before ablation (18.8 × 17 × 22 mm). (B) Thyroid vascularity disappeared immediately after RFA during the ablation session. (C) One month after the first RFA, there was a reduction in tumor size (17.6 × 14.6 × 15.4).

An 83‐year‐old male with a nodule in the left thyroid lobe was observed at a medical facility in Iran in October 2023. Ultrasonographic evaluation of the nodule revealed hypoechoic features with vascularity and significant dimensions of 18.8 × 17 × 22 mm. It contained some coarse calcification foci. These features categorized it as American College of Radiology Thyroid Imaging, Reporting, and Data System (ACR TIRADS) 4 (Figure 1A). The initial cytology from FNA revealed atypia of undetermined significance (AUS). Given that core needle biopsy has been shown to be safe in small nodules located in high‐risk areas when preceded by hydrodissection, as demonstrated in previous studies, the procedure was similarly performed in this patient using the same technique [10]. The core needle biopsy histology and immunohistochemical (IHC) staining results were consistent with MTC. IHC staining was positive for TTF1, calcitonin, synaptophysin, and chromogranin. Thyroglobulin was not detected in the tumor. These findings were supported by elevated serum calcitonin levels of 1216 pg/mL. A spiral computed tomography scan revealed no metastasis in the neck, chest, or abdomen.

In the ultrasonographic assessment of the neck, the tumor was confined to the left thyroid lobe without extracapsular extension or neck lymphadenopathy. Surgical intervention was recommended, but the patient refused and preferred to perform a less invasive procedure instead of surgery. Due to advanced age, comorbidities, and the patient's unwillingness to undergo surgery, we were compelled to perform ablation. Informed consent was obtained from the patient included in the study before RF ablation. For the procedure, we used a cooling system and manual settings. After local anesthesia with 3 mL of 2% lidocaine, the thyroid tumor under color Doppler sonography was ablated with a 7 mm active tip RF needle using a moving shout technique. Energy delivery began at 20 watts and was increased to approximately 35 watts. To prevent calcitonin release during ablation, we employed two specific techniques: (1) The needle was advanced close to the nodule while the generator remained off. Thermal energy was initiated only at the moment of needle entry into the nodule, minimizing stimulation. (2) We aimed to reduce the number of punctures to the lowest possible extent. We considered an area necrotic based on the echogenicity of the needle tip. Our procedural endpoint was the appearance of a continuous, peripheral echogenic line surrounding the entire nodule, indicating adequate ablation coverage. To mitigate potential complications, we performed extensive hydrodissection using approximately 150 cc of dextrose solution. The total procedure time was 9 min, and the time of energy delivery was 4 min (Figure 1B). Vocal cord function was monitored during the procedure. No complications were observed after the procedure. The patient was under observation after the procedure for probable side effects, and he was discharged in good condition.

One month later, ultrasonography revealed the avascular thyroid nodule. The size decreased from 18.8 × 17 × 22 mm in the pretreatment state to 17.6 × 14.6 × 15.4 mm at 1 month after ablation (3.6–2 mL). No new thyroidal lesions or lymphadenopathy were detected in this workup. The serum calcitonin concentration decreased to 768 and 14.8 pg/mL during 1 and 3 months of follow‐up after ablation, respectively. Six months after the ablation, it started to increase to 27.5 pg/mL and tumor sizes were 13 × 10 × 10 mm (0.79 mL); so, we decided to perform a second RFA. The ablation was performed the same as the first time; however, it lasted a shorter time (total time of 7 min and energy delivery time of 3 min) and it needed a lower serum (100 cc) for hydrodissection.

**FIGURE 2:**
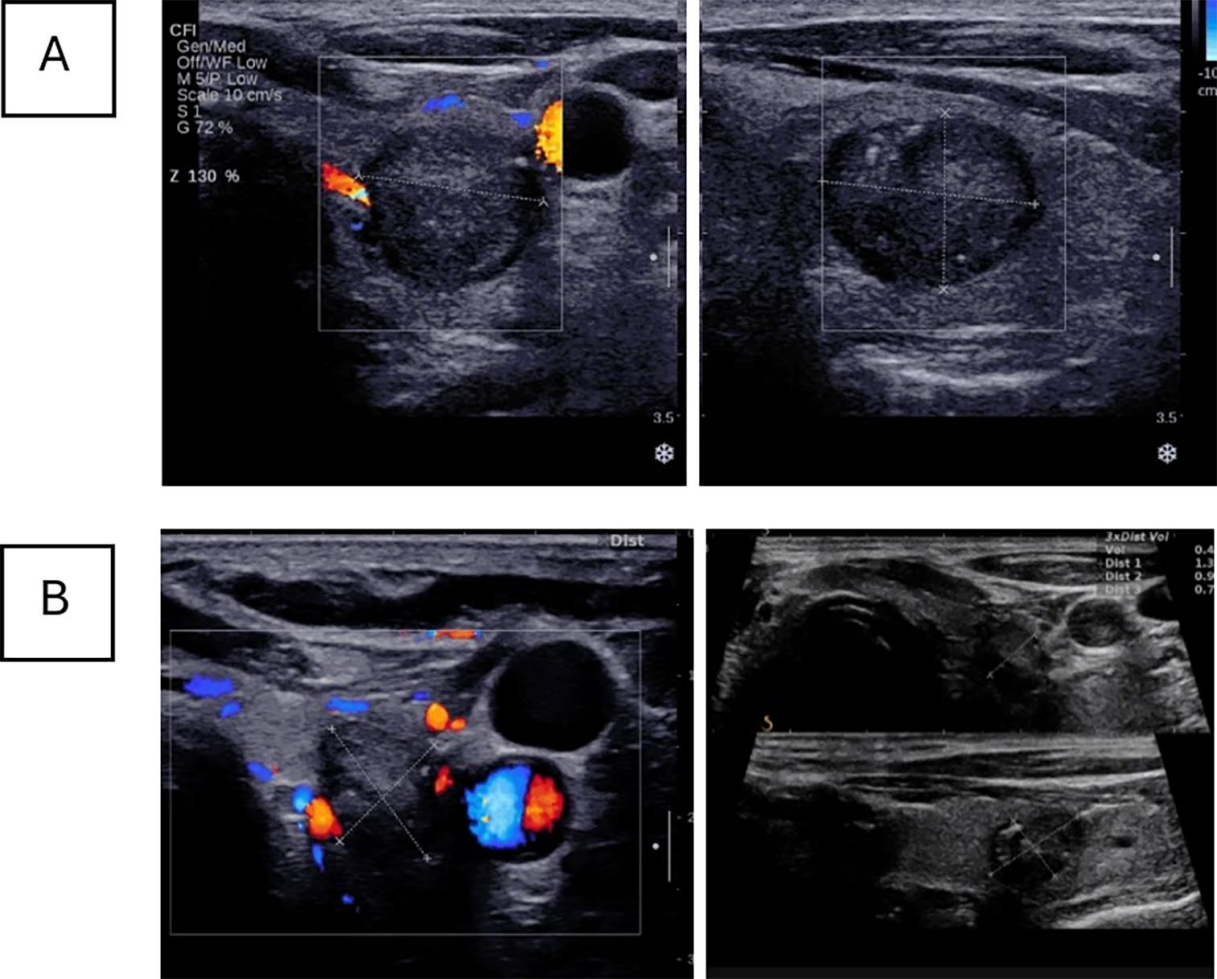
(A). Before the second ablation (6 months after the first RFA), there was a reduction in tumor size (13 × 10 × 10 mm). (B) Three months after the second ablation (9 months after the first RFA), the size of the thyroid nodule was 7.7 × 9 × 13.6 mm (0.49 cc) in thyroid ultrasonography.

**FIGURE 3:**
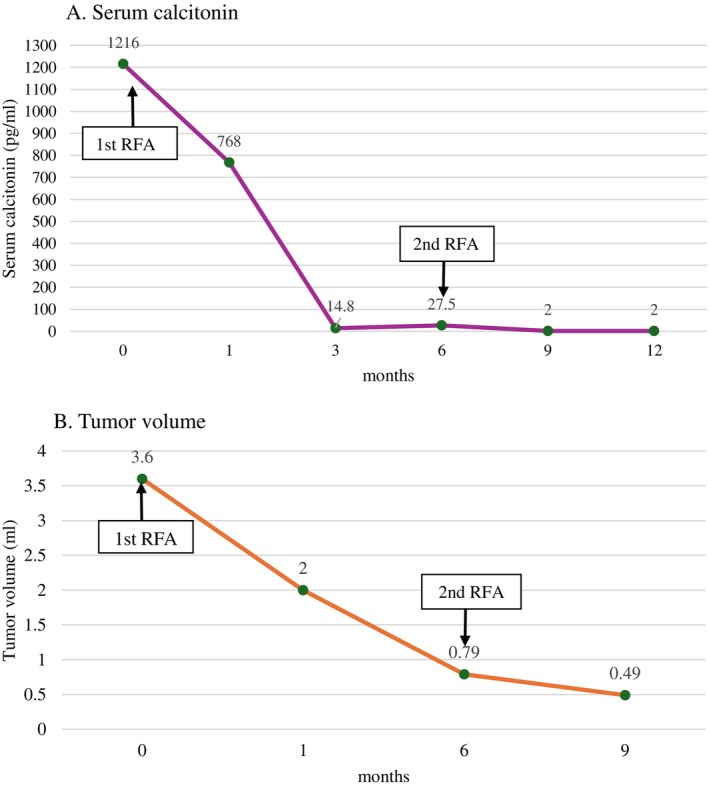
Changes in serum calcitonin (A) and tumor volume (B) during follow‐up after RFA. The serum calcitonin level significantly decreased from 1216 pg/mL preablation to less than 2 pg/mL over 1 year of follow‐up after RF ablation (A). The tumor volume reduction was remarkable during the follow‐up, as presented (B). RFA, radiofrequency ablation.

Nine months after the first RFA procedure, the patient's serum calcitonin level had decreased significantly to less than 2 pg/mL, and the sizes of the thyroid tumor were 7.7 × 9 × 13.6 mm (0.49 mL), and the tumor was stable biochemically and structurally over 6 months after the second ablation (Figure 2). Additionally, no lymphadenopathy in the neck was detected via ultrasonography after 1 year. The patterns of changes in serum calcitonin levels and tumor size during follow‐up are summarized in Figure 3. The patient did not report any complications, such as bleeding, infection, or hoarseness, during the follow‐up period.

## 3 Discussion

In the present case, the patient experienced an impressive 88% reduction in tumor volume, leading to a decrease in his calcitonin level to less than 2 pg/mL during the 1‐year follow‐up. The literature review indicates that most studies have focused on the effect of thermal ablation such as RFA on benign thyroid nodules and low‐risk papillary thyroid carcinoma and recurrent thyroid carcinoma in patients who refuse or are ineligible for surgery [11, 12, 13, 14, 15, 16]. RF ablation is an effective treatment modality for benign thyroid nodules, with significant volume reduction and symptom relief reported [17, 18].

In management of MTC, aggressive surgery remains the treatment of choice. In benign thyroid nodules, both surgery and RFA are safe, but RFA is associated with fewer complications and minimal pain. However, it does not allow for pathological examination, and in some surgical cases, hidden malignancies have been detected [19]. For papillary thyroid microcarcinoma, RFA offers less invasiveness, fewer complications, faster recovery, and better quality of life compared with surgery, though strict patient selection is essential [20]. In recurrent thyroid cancer, RFA and other ablation techniques have shown similar or better recurrence free survival than surgery at 1–6 years of follow up, with lower complication rates, and may be particularly effective for large tumors in palliative settings [21].

Most studies have discussed the efficacy of RFA for neck recurrent MTC after primary surgical resection [8]. Only a handful of case reports have evaluated RFA for early MTC in patients ineligible for surgery or for patients with a regional recurrence after surgical resection of the tumor [12]. To our knowledge, only one case report has evaluated the efficacy of RF ablation for nonmetastatic MTC. The authors reported a significant decline in serum calcitonin level with fibrotic tissue in place of the tumor over 15 months of follow‐up [6].

We followed the patient according to the serum calcitonin level and ultrasonographic features. In this case, the absence of lymphadenopathy and significant reduction in calcitonin levels suggest effective disease control. The serum calcitonin level and tumor volume represent high‐impact prognostic parameters in MTC management since they reflect disease progression and provide the basis for the prediction of patient outcomes. Higher concentrations of calcitonin correlate with larger tumor sizes and thus an increased risk of lymph node involvement, which has a significant effect on survival. Furthermore, larger tumors typically indicate more advanced cancer and would therefore worsen prognosis. Overall, the serum calcitonin concentration and tumor volume are very important in the risk stratification, management, and follow‐up of MTC [9]. Machens et al. highlighted that elevated serum calcitonin levels are indicative of a greater risk for metastases, necessitating ongoing surveillance [22]. Indeed, serum calcitonin levels greater than 1000 pg/mL are related to metastasis in lateral neck lymph nodes in 71% of cases [23]. In high calcitonin level (more than 500 pg/mL), surgery and systemic therapy are recommended; however, cases of calcitonin more than 500 were successfully ablated with RFA [6].

The patient's initial nodule size and the absence of vascularity postablation might reflect favorable prognosis indicators, warranting that future studies quantify these relationships further. The predictive factors for successful RF ablation outcomes have also been assessed in other studies. Cesareo et al. noted that nodule characteristics, including size and vascularity, can influence treatment efficacy [24]. There were no complications in our patient after the procedure. The same results were reported in other studies using RFA in thyroid nodules [8]. In this regard, Tong et al. presented a case report of recurrent MTC treated with RFA. They observed that RFA is not associated with any complications in follow‐up [25]. According to the literature review, fewer complications are associated with RFA than with surgery overall. However, the prevalence of complications depends on the expertise of the surgeon [26]. The results are summarized in Table 1.

**TABLE 1:** Complications of RFA in comparison with known complications of thyroidectomy.

Complication	RFA rate (%)	Thyroidectomy rate (%)
RLN injury	1.02	5–11
Hemorrhage	Low^a^	Moderate (requires monitoring)
Hypoparathyroidism	Rare	20–30
Infection	Low	Moderate
Hypothyroidism	Rare	Common

Abbreviation: RLN, recurrent laryngeal nerve.

^a^ Hemorrhage: although generally low, severe bleeding can occur post‐RFA, especially if large vessels are nearby.

We can hypothesize that some criteria can be considered for RFA in patients with MTC, such as tumor confinement to the thyroid, absence of lymphadenopathy and extrathyroidal extension, patient refusal or contraindication to surgery, and elevated but nonmetastatic calcitonin levels. However, since this is a single case report, generalizability is inherently limited. This finding requires validation through larger, controlled studies with longer duration of follow‐up. Additionally, comparative studies on RF ablation versus traditional surgical approaches, “wait and see” approach, and other nonsurgical treatments for MTC are warranted to better understand the benefits and risks associated with each treatment modality, especially with respect to patient quality of life. As the landscape of thyroid cancer treatment evolves, integrating these insights into practice will be crucial for optimizing patient outcomes.

In conclusion, RFA can be an alternative management method for MTC patients who are not candidates for surgery or who refuse it. It can result in significant reductions in nodule size and calcitonin levels with improvement in quality of life. However, prospective and larger studies will be needed and future research should focus on long‐term outcomes associated with RFA compared to traditional surgical methods, including recurrence rates, overall survival, and quality of life metrics.

## Author Contributions

**Hojat Ebrahiminik:** conceptualization, methodology, data curation, investigation, validation, supervision, visualization, resources, writing – review and editing. **Hossein Chegeni:** conceptualization, methodology, data curation, supervision, resources, validation, visualization, investigation, project administration. **Haleh Chehrehgosha:** conceptualization, investigation, methodology, validation, visualization, writing – review and editing, writing – original draft, data curation, formal analysis.

## Funding

The authors have nothing to report.

## Ethics Statement

All procedures performed in studies involving human participants were in accordance with the ethical standards of the institutional and/or national research committee and with the 1964 Helsinki Declaration and its later amendments or comparable ethical standards.

## Consent

All authors agree to publication.

## Conflicts of Interest

The authors declare no conflicts of interest.

## Data Availability

The data that support the findings of this study are available on request from the corresponding author. The data are not publicly available due to privacy or ethical restrictions.
